# Genomic and transcriptomic analysis of a library of small cell lung cancer patient-derived xenografts

**DOI:** 10.1038/s41467-022-29794-4

**Published:** 2022-04-19

**Authors:** Rebecca Caeser, Jacklynn V. Egger, Shweta Chavan, Nicholas D. Socci, Caitlin Byrne Jones, Faruk Erdem Kombak, Marina Asher, Michael H. Roehrl, Nisargbhai S. Shah, Viola Allaj, Parvathy Manoj, Sam E. Tischfield, Amanda Kulick, Maximiliano Meneses, Christine A. Iacobuzio-Donahue, W. Victoria Lai, Umeshkumar Bhanot, Marina K. Baine, Natasha Rekhtman, Travis J. Hollmann, Elisa de Stanchina, John T. Poirier, Charles M. Rudin, Triparna Sen

**Affiliations:** 1 Department of Medicine, Memorial Sloan Kettering Cancer Center, New York, NY 10065 USA; 2 Bioinformatics Core, Memorial Sloan Kettering Cancer Center, New York, NY 10065 USA; 3 Precision Pathology Center, Memorial Sloan Kettering Cancer Center, New York, NY 10065 USA; 4 Marie-Josée and Henry R. Kravis Center for Molecular Oncology, Memorial Sloan Kettering Cancer Center, New York, NY USA; 5 Antitumor Assessment Core Facility, Molecular Pharmacology Program, Memorial Sloan Kettering Cancer Center, New York, NY10065 USA; 6 Department of Pathology, Memorial Sloan Kettering Cancer Center, New York, NY USA; 7 Human Oncology and Pathogenesis Program, Memorial Sloan Kettering Cancer Center, New York, NY USA; 8 Perlmutter Cancer Center, New York University Langone Health, New York, NY USA; 9 Molecular Pharmacology Program, Memorial Sloan Kettering Cancer Center, New York, NY 10065 USA

**Keywords:** Cancer models, Small-cell lung cancer, Cancer genomics

## Abstract

Access to clinically relevant small cell lung cancer (SCLC) tissue is limited because surgical resection is rare in metastatic SCLC. Patient-derived xenografts (PDX) and circulating tumor cell-derived xenografts (CDX) have emerged as valuable tools to characterize SCLC. Here, we present a resource of 46 extensively annotated PDX/CDX models derived from 33 patients with SCLC. We perform multi-omic analyses, using targeted tumor next-generation sequencing, RNA-sequencing, and immunohistochemistry to deconvolute the mutational landscapes, global expression profiles, and molecular subtypes of these SCLC models. SCLC subtypes characterized by transcriptional regulators, *ASCL1, NEUROD1* and *POU2F3* are confirmed in this cohort. A subset of SCLC clinical specimens, including matched PDX/CDX and clinical specimen pairs, confirm that the primary features and genomic and proteomic landscapes of the tumors of origin are preserved in the derivative PDX models. This resource provides a powerful system to study SCLC biology.

## Abstract (web summary)

Creating accurate models of small cell lung cancer is essential to ensure the clinical relevance of results. Here, the authors create patient derived xenograft models from 33 patients and show, through multi-omics sequencing, that these models retain the primary features of the original.

## Introduction

Lung cancer is the leading cause of cancer death in men and women, responsible for more deaths than colon, breast, and prostate cancers combined^1^. Small cell lung cancer (SCLC) is an aggressive neuroendocrine (NE) tumor that accounts for ~15% of all lung cancers^2,3^. Most patients have metastatic disease at the time of diagnosis, a condition associated with a 5-year survival of <5%^4^. For decades, chemotherapy was the only treatment option for this disease. Immune checkpoint blockade (ICB) has proven effective for only a small subset of patients, whether the administered regimen targets the PD-1/PD-L1 axis alone or is combined with anti-CTLA-4^5^. ICB plus chemotherapy has been FDA-approved for the frontline treatment in SCLC based on the results of phase III ImPower133 trial, which demonstrated a modest but significant increase in overall survival and progression-free survival with the addition of atezolizumab to chemotherapy^6^. More recently, results from the CASPIAN trial led to the FDA approval of another anti-PD-L1 antibody, durvalumab, based on similarly modest but clinically meaningful survival benefit^7,8^. Despite the emergence of novel targeted therapies, including inhibitors of PARP, EZH2, and BCL2, their clinical benefits have been limited, and have not led to regulatory approvals. Relative to other types of lung cancer, therapeutic options in SCLC are limited and offer only transient benefits for the large majority of patients.

A major challenge to identifying effective treatments has been the relative lack of model systems that accurately reflect SCLC tumorigenesis. Access to clinically relevant tissue is limited because surgical resection is rare in metastatic SCLC. Until recently this has made researchers heavily reliant on established cell lines and genetically engineered mouse models (GEMM), which both have inherent limitations. Cell lines often carry a biased mutational repertoire and decades of in vitro growth further selects these lines. The available SCLC cell lines were largely generated before the advent of the current standard of care combination therapies and may not accurately represent current resistance mechanisms. GEMMs generally fail to recapitulate the diverse mutational landscape caused by years of tobacco use^9^. Patient-derived xenografts (PDX) and circulating tumor cell-derived xenografts (CDX) have emerged as valuable tools to interrogate the genomic landscape of SCLC^9–12^. These patient-derived models have their own limitations, most notably the requirement to be maintained in an immunosuppressed murine host, precluding assessment of novel therapies to stimulate the adaptive immune system. This limitation is counterbalanced by several advantages—notably proximity to primary patient tumors, and avoidance of the selective pressures associated with establishing clonal cell lines able to grow in the in vitro tissue culture environment^13^. Observed phenotypes in the PDX sample can be directly traced back to the patient and correlated with clinical response to therapies^10^. SCLC patients typically have a high prevalence of circulating tumor cells, which has been used as a biomarker of tumor burden during treatment^14^. Invasive SCLC tumor biopsies and surgical resections can be avoided by the collection of circulating tumor cells in the blood for CDX modeling^15,16^.

SCLC subtypes were recently characterized and defined by the relative expression of genes encoding four major transcriptional regulators, *ASCL1*, *NEUROD1*, *POU2F3*, and *YAP1* (defining subtypes SCLC-A, SCLC-N, SCLC-P, and SCLC-Y, respectively)^17^. Since then, the subtype-defining validity of *YAP1* has been questioned, and SCLC-Y is now also described by an “inflamed” gene signature (SCLC-I)^12^. SCLC-A remains the most common subtype (~50%), followed by SCLC-N (~20%), SCLC-Y (~20%), and SCLC-P (10%)^12,18^. Reflecting the clinical landscape, all previous publications have only reported data on PDXs/CDXs belonging to SCLC-A and SCLC-N subtypes. The rare non-NE SCLC-P subtype has not been reported as a PDX model and therefore has been less extensively characterized.

Here, we present a resource comprised of a cohort of 46 extensively annotated PDX/CDX models derived from 33 patients with SCLC. We perform multi-omic analyses, using targeted tumor next-generation sequencing (NGS) by MSK-IMPACT, RNA-sequencing, and immunohistochemistry (IHC) to deconvolute the mutational landscapes, global expression profiles, and molecular subtypes of these SCLC models. We directly compare these genomic and proteomic profiles to a subset of SCLC clinical specimens, including matched PDX/CDX and clinical specimen pairs. Moreover, we were able to establish and deeply characterize ten PDX models from one primary and nine metastatic tumors from an individual whose SCLC tumor belongs to the POU2F3 subtype. Therefore, our study provides insight into the genomic, transcriptomic, and protein landscape in PDX/CDX models of the different subtypes of SCLC, including SCLC-P.

## Results

### Generation of the PDX/CDX panel

**Fig. 1:**
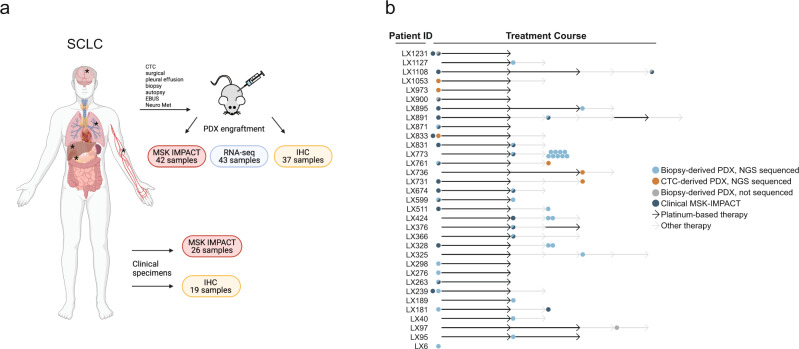
Generation of the PDX/CDX panel. **a** Overview of the patient-derived/circulating tumor cell-derived xenograft (PDX/CDX) cohort and clinical specimens. Samples were collected from surgical, pleural effusion, biopsy, autopsy, EBUS, Neuro Met, and CTC isolation and either engrafted into immunodeficient mice to generate PDX/CDX models or directly sent for targeted sequencing (MSK-IMPACT) and Immunohistochemistry (IHC). Created with BioRender.com. **b** Swimmers Plot depicting sample collection of the PDX/CDX cohort during treatment course (Platinum-based therapy— black arrow, Other therapy—gray arrow). Models derived from biopsies are annotated with a blue or gray dot (with next-generation sequencing [NGS] or not, respectively). Models derived from circulating tumor cells (CTC, sequenced with NGS) are annotated with an orange dot. Dark blue indicates NGS (MSK-IMPACT) for the clinical sample. Arrows are not to scale in terms of treatment duration.

Clinical characteristics of the 46 SCLC PDX/CDX models (33 patient donors) can be found in Supplementary Data 1. World Health Organization’s pathologic classification of SCLC includes two subtypes: SCLC (“pure” SCLC) and combined SCLC, with the latter demonstrating admixture with any histology of non-small cell lung cancer^19^. Our cohort included five cases representing combined SCLC: three with a large cell NE component, one with squamous cell carcinoma, and one with adenocarcinoma. Of the 46 models, 40 (87%) were tissue-derived PDXs and 6 (13%) were CDXs. The average tumor engraftment time was 112 days, with no appreciable difference between PDXs (110 days) and CDXs (131 days). Out of 198 pure SCLC and 29 mixed histology samples that were initially injected to create a PDX model, 45 samples (23.81%) and 13 samples (44.83%) were successfully engrafted, respectively. IHC was performed on 37 samples, RNA-seq on 43 samples, and NGS via MSK-IMPACT on 42 samples (Fig. 1a). The patient cohort was 55% (18/33) male and 45% female, with an average age of 66 years at diagnosis. Most patient donors, 27 (82%) of 33, were current or former smokers. Nineteen of 33 patients (58%) presented with extensive-stage (ES) disease while the remainder (14/33, 42%) had limited-stage (LS). Treatment history and a depiction of sample collection is shown in Fig. 1b, with most patients receiving cisplatin or carboplatin and etoposide therapy either immediately after, or prior to, sample collection for model generation. IHC and targeted NGS via MSK-IMPACT was available for 19 and 26 of the patient tumors from which PDXs/CDXs were derived, respectively (Fig. 1a).

### MSK-IMPACT targeted tumor sequencing of matched PDX and clinical SCLC samples

**Fig. 2:**
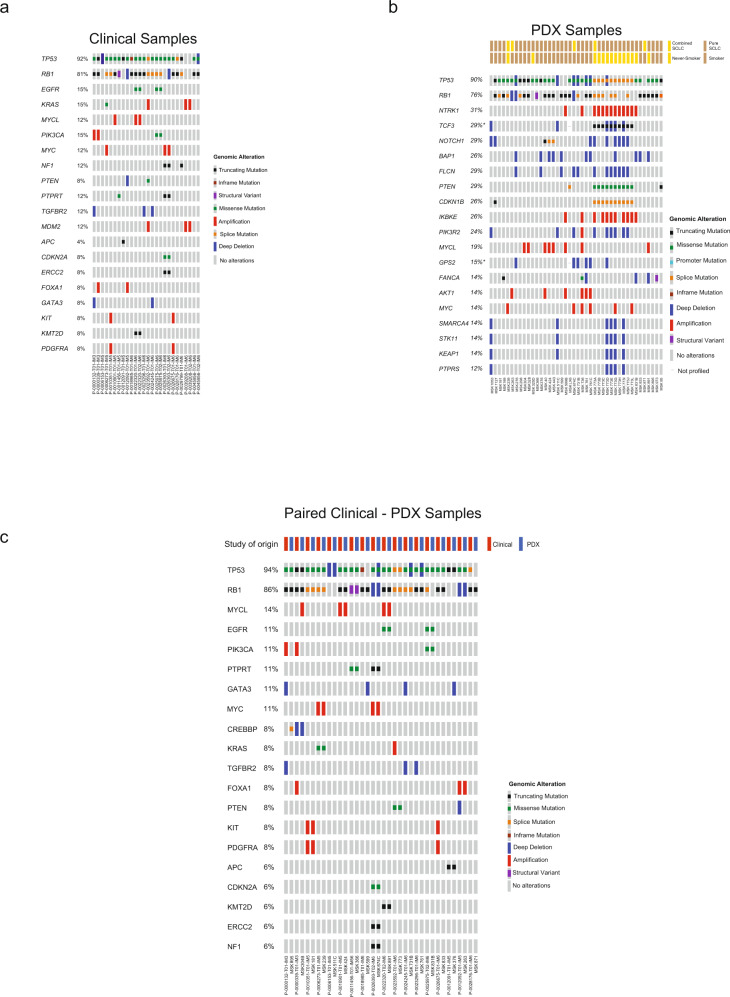
Matched PDX and clinical SCLC sample analysis. Genomic landscape in SCLC (**a** Clinical Samples, **b** patient-derived xenograft (PDX) Samples, **c** paired Clinical-PDX samples) samples representing putative driver alterations (i.e., excludes variants of unknown significance) in the top 20 most frequently altered genes as detected by MSK-IMPACT^20^ targeted next-generation sequencing.

We conducted comparative genomic analyses on our patient tumors versus PDX models to establish the genomic fidelity of the PDX models compared with patient tumor specimens collected at the same time point. A total of 42 PDX samples and 26 clinical samples were profiled by MSK-IMPACT targeted tumor sequencing^20–22^. Of those, 18 cases included matched pairs, and the top 20 altered genes in these cohorts are presented in Fig. 2. Consistent with known key characteristics of SCLC, NGS revealed mutations (primarily missense and truncating mutations) with the rarer splice and in-frame mutations in *TP53* in 24 out of 26 (92%) clinical samples and truncating splice mutations, structural variants, and deep deletions in *RB1* in 21 out of 26 (81%) clinical samples (Fig. 2a). Despite the rarity of MAPK pathway mutations in SCLC, two clinical samples (P-0039208-T01-IM6 and P-0039208-T02-IM6) carried *KRAS* amplifications without *TP53* and *RB1* mutations^23^. Notably, these samples also harbored *MDM2* amplification, which were only found in one other clinical sample out of 26. *MDM2* is a negative regulator of *TP53*, so amplification-induced overexpression could functionally inactivate *TP53*, potentially phenocopying missense/truncating *TP53* mutations in these samples. Four samples from patients that never smoked (samples P-0022320-T01/2-IM6 and P-0025975-T01/2-IM6,) demonstrated oncogenic mutations in *EGFR* consistent with a histologic transformation from lung adenocarcinoma^24^. Alterations in *PIK3CA, KMT2D*, and *SPEN* were also observed; and could be traced back to the matched clinical sample.

A higher density of alterations, particularly copy number changes, were identified in the genomic landscapes of the 42 PDX samples than in the clinical samples (Fig. 2b), consistent with the greater purity of human genomic DNA from cancer cells in the PDX (due to the absence of human stromal cells^25^). Mutations in *TP53* and *RB1* were largely retained (90% and 76% of samples, respectively). One patient (MSK773) from whom several PDXs were generated had PDX models consistently demonstrating splice site mutations in *TP53* and *CDKN1B* and a missense mutation in *PTEN*. MSK1053, MSK511c, and MSK773D/E/G/I samples harbored mutations in *SMARCA4, STK11, KEAP1*, and *PTPRS* (except MSK511c); mutations more commonly observed in non-small cell lung cancer^26^^-^^27^.

For 18 cases, paired clinical-PDX samples were available for direct comparison to determine whether PDX samples retained features of the original patient tumors (Fig. 2c). Most (89%, 16/18) pairs retained genomic alterations in *TP53* and *RB1*. The PDXs derived from MSK599 and MSK871 both lacked the *TP53* alterations found in the corresponding clinical sample. Besides *TP53* and *RB1*, most matched samples harbored the same additional mutations with a few samples exhibiting variability. For example, MSK304B lost *PIK3CA* and *FOXA1* amplification in the PDX sample but gained an *MYCL* amplification. The observed differences might reflect the intrinsic clonal heterogeneity of the biopsies used for direct sequencing vs. PDX generation or could be due to bottlenecking and selection in PDX generation; these possibilities are not mutually exclusive. Overall, these results demonstrate that primary features of the genomic landscapes of the tumors of origin were preserved in the derivative PDX models.

### SCLC subtype and NE markers

**Fig. 3:**
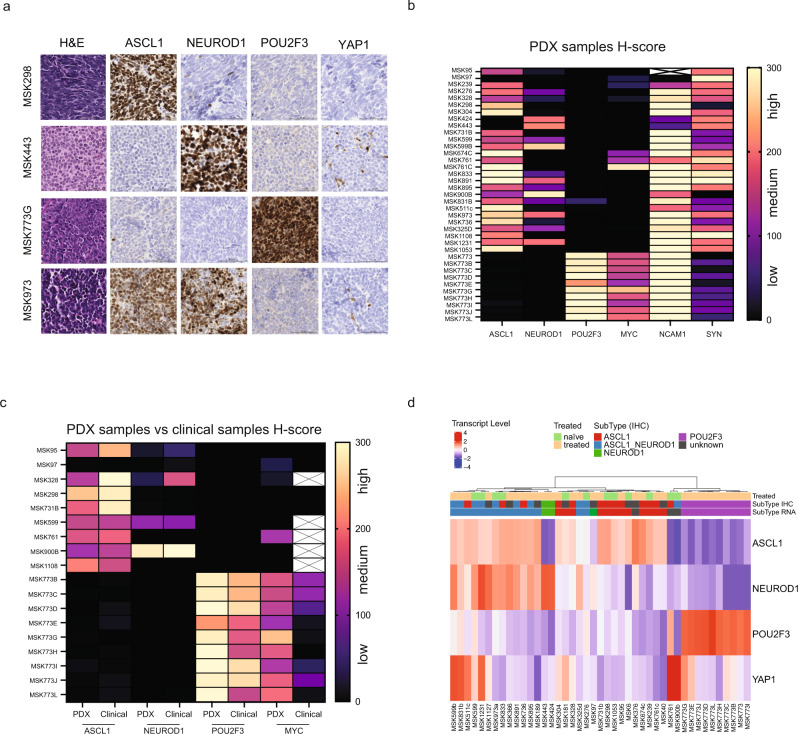
SCLC subtype annotation and neuroendocrine markers. **a** H&E and immunohistochemistry images for the indicated proteins are shown. Scale bar, 50 μM. Four representative tumors are shown. Three independent cores per sample were stained for H&E and the indicated protein with a representative example shown here. **b** Heatmap depicts H-scores derived from 37 scored patient-derived xenograft (PDX) tumors for the fraction of positive cells for each protein shown, as well as staining intensity. White box with a cross (MSK95, NCAM1) indicates staining failed. Source data are provided as a Source Data file. **c** Heatmap depicts H-scores derived from 18 paired PDX tumors and clinical samples for the fraction of positive cells for each protein shown, as well as staining intensity. White boxes with cross indicate no staining available. Source data are provided as a Source Data file. **d** Heatmap of gene expression of 43 PDX samples for the selected markers; ASCL1, NEUROD1, POU2F3, and YAP1. Color-coded panel on top depicts treatment status (naïve or treated) and subtype annotation based on immunohistochemistry (IHC) or RNA-sequencing. Source data are provided as a Source Data file.

SCLC diagnoses of donor patient biopsies were morphologically confirmed by a pathologist. Selected cases exhibited spindle cell and nesting growth patterns, along with the predominance of sheet-like/solid morphology. Representative examples of SCLC PDX histology by hematoxylin and eosin (H&E) staining are shown in Fig. 3a. SCLC has been divided into biologically distinct subtypes based on the expression of the transcriptional regulators *ASCL1, NEUROD1, POU2F3*, *and YAP1*^17^. However, recent reports show only sporadic expression of *YAP1* in clinical samples and CDXs^28,29^, questioning the role of *YAP1* in SCLC. We performed IHC on 37 PDX/CDX samples, and a pathologist reviewed and scored tumors for the fraction of positive cells for each protein, as well as staining intensity (*H*-score). In our prior analysis of primary human tumors by IHC, it was noted that many SCLC tumors co-express ASCL1 and NEUROD1, defining an intermediate between dichotomous expression of these factors^28^. Figure 3a shows representative staining of four selected cases derived from *ASCL1, NEUROD1*, *POU2F3*, and *ASCL1/NEUROD1* subtypes (see Supplementary Figs. 1–4 for other cases). Most PDXs showed medium/high *H*-score expression for *ASCL1, NEUROD1*, or both. A distinct subset of ten PDXs (MSK773, B-L) derived from one patient (biopsy and different tissue sites post-mortem) were strongly positive for *POU2F3* and negative for the other subtype-defining transcription factors *(*Fig. 3a, b). *YAP1* expression was consistently absent or very low across all samples, regardless of intracellular localization (nuclear or cytoplasmic staining, Supplementary Fig. 5a). This parallels the recent studies noted above^28,29^, further suggesting that *YAP1* may not be an optimal marker to distinguish between SCLC subtypes.

Consistent with previous literature, all *POU2F3*-expressing PDXs also expressed *MYC* (Fig. 3b and Supplementary Figs. 1–4). Most PDXs (with the exception of 5) showed high expression of the NE marker NCAM1; most also expressed synaptophysin, although this was lower or absent in the *POU2F3* PDXs (Fig. 3b and Supplementary Figs. 1–4). Paired samples, with analysis of a clinical sample and the corresponding PDX, were available for 18 cases, and H-scores of subtype markers ASCL1, NEUROD1, POU2F3, and YAP1, as well as expression of MYC, were similar or unchanged in most pairs (Fig. 3c and Supplementary Figs. 5B,  6, 7). These data illustrate that the PDX cohort largely retains both the genomic and proteomic features of the original SCLC biopsy samples, increasing confidence in this model system as a platform for studying SCLC.

The expression of subtype markers was further confirmed by RNA-seq (Fig. 3d). Heatmap shows samples clustered based on subtype expression (*ASCL1, NEUROD1, POU2F3*, and *YAP1*). Subtype annotation based on IHC and RNA-seq largely overlapped, and no pattern emerged between naïve and treated samples. Interestingly, the strong *YAP1* RNA expression seen in some PDXs (MSK599b, MSK831b, MSK511c, MSK773G, MSK1231, MSK761, MSK900b, and MSK773E) was not observed on IHC (Supplementary Fig. 5C). Recent data have suggested that *YAP1*-driven SCLC cell lines express YAP together with its homologous heterodimeric transcriptional coactivator TAZ to both activate and induce negative feedback regulation of the Hippo pathway^30^. YAP1 subtype has also been correlated with NOTCH expression (*NOTCH1, NOTCH2*, and *NOTCH3*)^30,31^. We explored *YAP/TAZ* and their downstream transcriptional targets in our PDX/CDX dataset (Supplementary Fig. 5D). Strikingly, the *POU2F3*-positive PDX subset highly expressed YAP1 downstream genes, including *NOTCH2* and *NOTCH3*, regardless of whether *YAP1* itself was expressed. *ASCL1*- and *NEUROD1*-driven PDXs had lower expression of such genes, suggesting this pathway is inactivated in the NE subsets as previously described^30^. Notably, two samples, SCLC-A/N MSK900b and SCLC-A MSK761, showed relatively high expression of *YAP1*, similar to the SCLC-P models, however, YAP1 protein staining was absent (Supplementary Fig. 5C, D).

Recently, SCLC lacking high expression of ASCL1, NEUROD1, and POU2F3 have been re-categorized on the basis of their inflamed gene signature, including immune checkpoint genes and human leukocyte antigens (HLAs), rather than solely based on the expression of *YAP1*^12^. This prompted us to look at the expression of HLAs and antigen-presenting gene signatures^12^ in our PDX cohort. Seven of 43 models (MSK831b, MSK511c, MSK325d, MSK773B, MSK773H, MSK761, and MSK891) showed high expression of HLAs and related antigen presentation genes such as HLA-DRA or HLA-DBP1 (Supplementary Fig. 5e); these models were diverse and were not clearly attributed to a distinct subtype or treatment status (naïve/treated).

### Transcriptional analysis of SCLC tumors of different subtypes

**Fig. 4:**
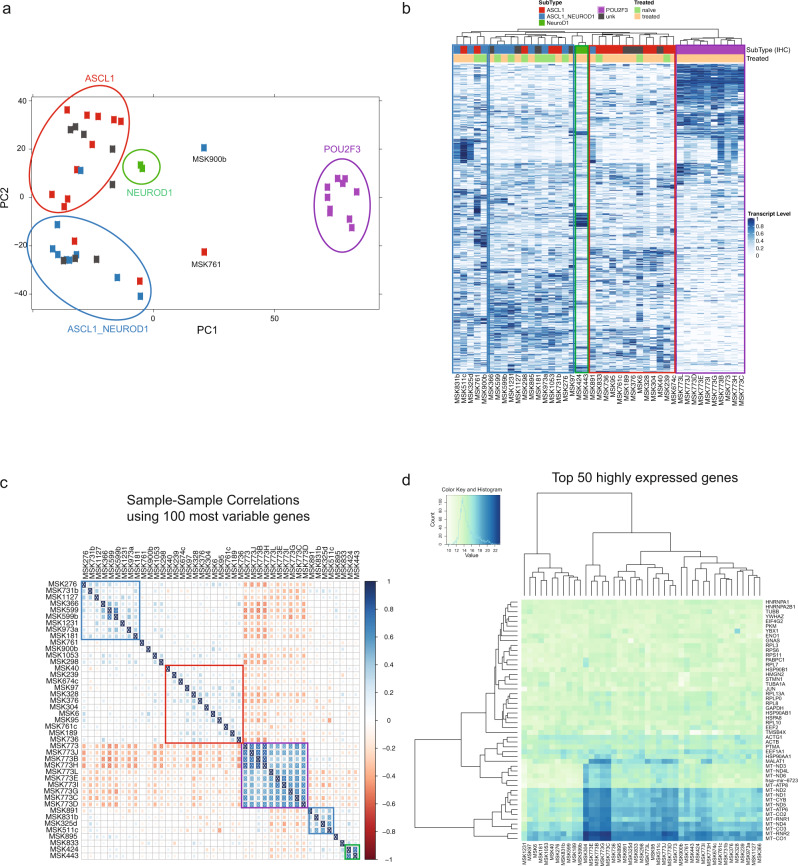
Transcriptional profile analysis of SCLC tumors of different subtypes. **a** Principal component analysis plot showing 43 patient-derived xenograft (PDX) samples color-coded based on immunohistochemistry (IHC) subtype annotation. Circles indicate four separate clusters (*ASCL1*-driven cluster, red; *ASCL1/NEUROD1*-driven cluster, blue; *NEUROD1*-driven cluster, green; *POU2F3*-driven cluster, purple). Source data are provided as a Source Data file. **b** Unbiased hierarchical clustering of 43 PDX samples. Color-coded panel on top depicts treatment status (naïve or treated) and subtype annotation based on IHC. Boxes indicate five separate clusters (two *ASCL1/NEUROD1*-driven clusters, blue; one *NEUROD1*-driven cluster, green; one *ASCL1*-driven cluster, red; one *POU2F3*-driven cluster, purple). Source data are provided as a Source Data file. **c** Sample-sample correlation plot using the top 100 highest variance genes. The Spearman correlation was used and the samples were ordered using hierarchical clustering with complete linkage. Five clusters emerged and are boxed (two *ASCL1/NEUROD1*-driven clusters, blue; one *NEUROD1*-driven cluster, green; one *ASCL1*-driven cluster, red; one *POU2F3*-driven cluster, purple). Source data are provided as a Source Data file. **d** Heatmap of the top 50 highly expressed genes in 43 PDX samples. Blue indicates high expression and green indicates low expression. Color key and histogram represents the normalized log 2-transformed counts. Source data are provided as a Source Data file.

To compare the SCLC tumors of different subtypes, we applied principal component analysis (PCA) to the top 500 genes ranked by variance over all samples (Fig. 4a). SCLC-P tumors clustered closely together and were distinct from the non-SCLC-P tumors. SCLC-A and SCLC-A/N formed separate clusters with some overlap. The two SCLC-N tumors lacking ASCL1 clustered closely together, and tumors with no subtype annotation based on IHC clustered near SCLC-A or SCLC-A/N cohorts. Notably, the two samples (SCLC-A/N MSK900b and SCLC-A MSK761, annotated in Fig. 4a) with *YAP1* downstream gene expression patterns similar to SCLC-P samples were positioned in an intermediate space between non-SCLC-P and SCLC-P samples, in relatively close proximity to the SCLC-P samples. These two PDXs also clustered closely together, but distinct from the SCLC-P samples, in hierarchical clustering (Fig. 4b). SCLC-A/N MSK900b and SCLC-A MSK761 were both chemonaïve, and all SCLC-P samples were treated. Overall, treatment status did not appear to be a determinant of clustering (Fig. 4b).

Unbiased hierarchical clustering revealed distinct clusters, suggesting one clear cluster to be of *POU2F3* origin (purple box), one cluster of mostly *ASCL1* origin (red box), one cluster of *NEUROD1* origin (green box), and two separate clusters of mostly *ASCL1/NEUROD1* or *ASCL1* origin (blue boxes) based on IHC (Fig. 4b). These suggested clusters were largely preserved when the analysis was based on the 100 most variable genes (Fig. 4c), with a few samples not falling into any cluster or into a different cluster than shown in Fig. 4b. However, clusters changed slightly when analyzing samples based on the top 50 highly expressed genes with only a subset of the SCLC-P samples forming a clear cluster (Fig. 4d). This subset of SCLC-P was notable for highly expressed mitochondrial genes such as *MT-RNR2* or *MT-CO3/1*. In summary, PCA analysis and hierarchical clustering revealed distinct subtypes that were primarily reflective of the relative expression of *ASCL1, NEUROD1*, *and POU2F3*, including a substantial population with co-expression of *ASCL1* and *NEUROD1*.

### Gene expression signatures in SCLC

**Fig. 5:**
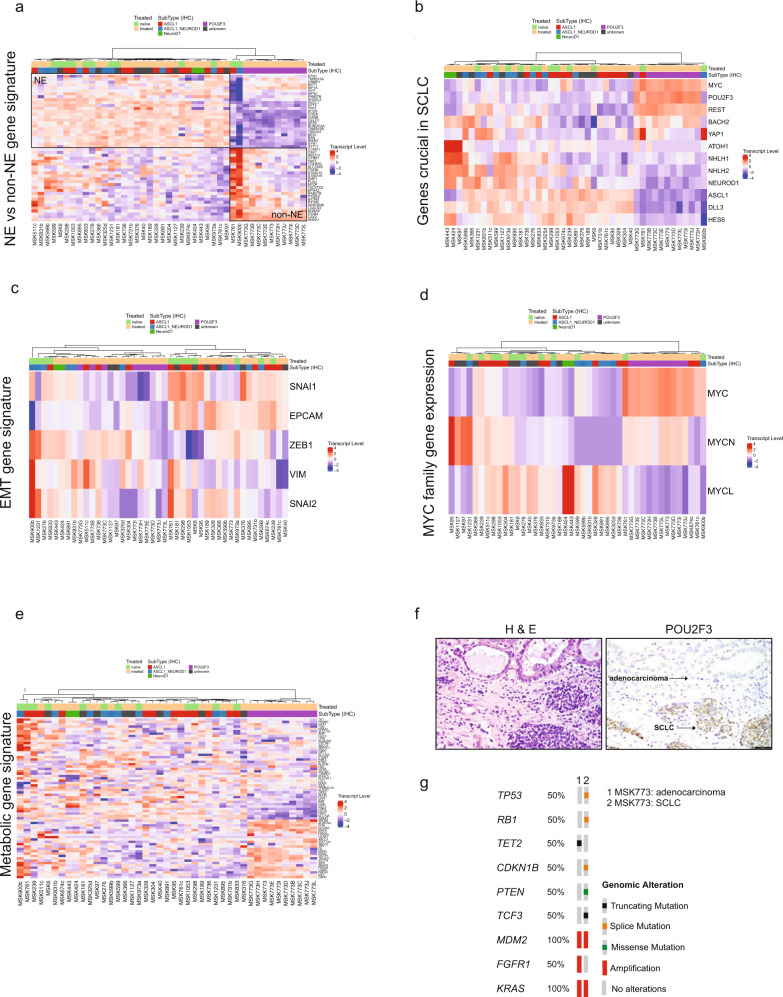
Gene expression signatures in SCLC. Heatmap of gene expression of 43 patient-derived xenograft (PDX) samples for the selected gene signatures; neuroendocrine (NE) vs. non-NE gene signature^32^ (**a**), genes crucial in SCLC (**b**), EMT gene signature (**c**), MYC family gene expression (**d**), and metabolic gene signature^42^ (**e**). Color-coded panel on top depicts treatment status (naïve or treated) and subtype annotation based on IHC. Source data are provided as a Source Data file. **f** H&E and immunohistochemistry image for POU2F3 are shown for the clinical MSK773. Scale bar as indicated, 20 μM. **g** Genomic landscape in MSK773A (adenocarcinoma) vs. MSK773A (SCLC) samples representing putative driver alterations (i.e. excludes variants of unknown significance) in altered genes as detected by MSK-IMPACT^20^ targeted next-generation sequencing assay.

To differentiate lung NE cells from non-NE cells, we assessed these PDX/CDX models using a previously defined 50-gene NE signature tool^32^. As expected, NE subtypes (SCLC-A, SCLC-N, and SCLC-A/N) were markedly different from non-NE (SCLC-P) subtypes (Fig. 5a). The two non-SCLC-P samples that clustered closest to the SCLC-P group in PCA (Fig. 4), MSK761 (SCLC-A) and MSK900b (SCLC-A/N), here showed the most distinctive expression of genes in the cohort, with high expression of the non-NE determinant genes and strikingly low transcription levels of NE genes. Genes that were highly expressed in the NE-expressing subset included *SYN1, INSM1*, and *CHGA*. Drilling down on a few genes highlighting NE transcriptional regulators also revealed high expression of *REST* and *BACH2* and low expression of *DLL3* and *ATOH1* in the non-NE SCLC-P subtype (Fig. 5b).

While SCLC is primarily an epithelial disease, epithelial to mesenchymal transition (EMT) has been associated with the non-NE subset of SCLC and is defined by expression of vimentin (encoded by *VIM*) and loss of epithelial cell adhesion molecule (*EPCAM*)^32,33^. SCLC-P samples did not express detectable *EPCAM* but showed variable (low to medium) expression of *VIM* as well as low expression of EMT transcription factors *SNAI1/2* and *ZEB1* (Fig. 5c). *EPCAM* and *SNAI1* expression was heterogenous but tended to be highest in SCLC-A. No clear SCLC subtype associations emerged from these genes or a more extensive EMT gene signature^34^ (Fig. 5c and Supplementary Fig. 8). These findings are consistent with a recent report describing low *VIM* expression in three CDXs of the non-NE subtype, suggesting that EMT is not always a hallmark of non-NE cells in SCLC^9^.

### MYC gene family member expression

*MYC, MYCL*, and *MYCN* define a gene family implicated in SCLC oncogenesis and subtype differentiation^35–37^. To determine the relative expression of MYC family genes, we analyzed bulk RNA-sequencing for *MYC, MYCN*, and *MYCL* and also compared this to MYC IHC (validated antibodies are unavailable for MYCN and MYCL)^9,38^ data in Fig. 3. Paralleling protein assessment of MYC, SCLC-P samples highly expressed *MYC* at the RNA level (Fig. 5d). These samples expressed *MYCN* to a lesser degree and *MYCL* was low or absent. Not surprisingly, SCLC-A/N MSK900b and SCLC-A MSK761, which have consistently clustered with the SCLC-P cohort, also expressed high levels of *MYC* but did not express *MYCN* or *MYCL*. Most of the SCLC-A samples showed expression of *MYCL*, consistent with the previous reports^39^. Interestingly, the two SCLC-N samples (MSK424 and MSK443) in the cohort had the highest *MYCL* transcript expression levels (Fig. 5d). MSK-IMPACT targeted tumor sequencing^20^ revealed an MYCL amplification for some samples that harbored high *MYCL* expression, including MSK424, MSK304, MSK40, MSK599b, MSK891, and MSK736. Recent reports highlight that SCLC subtypes expressing different MYC family members are metabolically distinct^40,41^. We proceeded to look at a metabolic gene signature^42^ and interestingly, SCLC-P samples showed high expression of a subset of metabolic genes in comparison to the non-SCLC-P samples (Fig. 5e) such as *ABCB6, PGD*, or *G6PD*. While a clear subset of genes was overexpressed in the *MYC*^hi^ SCLC-P samples, no clear set of metabolic genes was regulated in the *MYCN*^hi^ or *MYCL*^hi^ SCLC samples, highlighting some degree of metabolic heterogeneity in our SCLC sample set.

### Origin of the SCLC-P models

About 10% of SCLC cases are driven by the transcription factor *POU2F3*; however, to date, there are no reports of an animal model representing this subtype. We were fortunate to obtain a set of *POU2F3*-driven PDXs, derived from a male patient in his 70s with ES-SCLC. This patient was a life-long never-smoker, unusual for SCLC, but he did have a history of Hodgkin’s disease over 40 years earlier, for which he had received mantle field radiation. The patient presented with a lung mass, bone metastases, and a large volume of pleural effusion, diagnosed by pleural fluid cytology as SCLC. He was initially treated with carboplatin and etoposide, and after initial response had radiological progression within 6 months of initial diagnosis. He underwent a percutaneous lung biopsy at that time, from which an initial PDX was established (MSK773). This biopsy showed an admixed SCLC and lung adenocarcinoma (Fig. 5f). RB immune expression was lost in the SCLC and retained in the adenocarcinoma. The resulting PDX demonstrated SCLC histology, strongly positive for POU2F3 (Fig. 3). The patient was subsequently treated with ipilimumab and nivolumab, without response. Prior to death, the patient elected to participate in our institutional medical donation program, allowing us to perform an immediate post-mortem autopsy, enabling the establishment of nine additional PDXs from recurrent and metastatic sites (lung, chest, liver, diaphragm, rib). As shown in Fig. 3, all ten samples from this patient were *POU2F3*-positive by IHC and RNA-sequencing and expressed *MYC*.

POU2F3 expression in the lung is normally tightly restricted to tuft cells, rare chemosensory epithelial cells^43^. The high expression and strong dependence on POU2F3 suggested that the SCLC-P subtype might arise through a malignant transformation of tuft cells^44^. Lung adenocarcinoma arises from the much more common type II pneumocyte or related progenitor cells^45^. It has also been extensively described that lung adenocarcinoma can undergo histologic transformation to NE SCLC, either de novo or as a mechanism of acquired resistance to targeted inhibitors^46–48^. The admixture of lung adenocarcinoma with this POU2F3-positive SCLC (Fig. 5f) implies that the type II pneumocyte might also serve as a precursor for SCLC-P. The mixed histology of this case suggested single cancer with differentiation into two lineage fates, or a “collision” tumor derived from independent transformation events in two distinct cells of origin. To distinguish between these possibilities, we performed MSK-IMPACT sequencing separately on the adenocarcinoma and SCLC components. Shared somatic mutations and copy number changes confirm clonality between these distinct histologies, with *RB1* and *TP53* loss specific to the SCLC (Fig. 5g). These data support that SCLC-P can derive from the same cell of origin as lung adenocarcinoma. The set of 10 MSK773 PDX from various anatomically distinct sites will serve as a particularly informative resource for understanding the biology and evolution of the rare SCLC-P subtype.

## Discussion

Existing model systems used to functionally interrogate the SCLC landscape include cell lines and GEMMs. Both model systems come with limitations; cell lines harbor a strong selection bias in the successful establishment of in vitro clonal growth, and GEMMs lack the diversity of mutations caused by long-term tobacco use. As such, other model systems that accurately recapitulate SCLC biology are needed. Here, we present in detail a large cohort of SCLC PDX/CDX models, representing different disease stages and treatment histories. We characterized molecular subtypes and the global gene expression landscapes of SCLC in our cohort using a multi-omic approach, including IHC, RNA-sequencing, and targeted tumor next-generation sequencing (by MSK-IMPACT).

All models presented were successful engraftments with an average time to first-generation tumor growth of 112 days. Models were derived from a variety of sources (e.g., biopsy, pleural effusion, blood) and tissue sites (e.g., lung, lymph node, liver, brain). PDX tumors largely retained clinical features of the original biopsy, including the hallmarks of SCLC, inactivation of *TP53* and *RB1*. The significance of the few differences we did observe in genomic spectra of primary tumors and their derivative models remains to be elucidated. A caveat is that these PDX/CDX models are maintained in immune-incompetent mice: the absence of an intact adaptive immune system and replacement of human stromal elements by murine cells may alter tumor-intrinsic features of antigen presentation and immune profiling.

Current first-line standards of care for SCLC include platinum-based chemoimmunotherapy for ES disease and chemoradiotherapy for LS disease. Recent reports have shown SCLC cell lines respond differently to drug treatment based on subtype; for example, SCLC-P cell lines appear to be sensitive to PARP inhibition^12^. Similarly, SCLC GEMM models suggest that MYC-high SCLC may have selective sensitivity to Aurora kinase inhibition^49^. With new therapies emerging from early clinical trials, it will be important to identify the SCLC subtypes of patients to validate these observations and to inform future treatment strategies. Consistent with the known distribution of subtypes, most of our samples express *ASCL1* or both *ASCL1* and *NEUROD1*. We also identified ten *POU2F3*-driven SCLC samples, derived from one donor. Characteristic features of these models include high POU2F3 and MYC expression by IHC and RNA-seq and low expression of NE markers. Unbiased hierarchical clustering by global gene expression largely recapitulates subtype assignments based on the primary transcription factors. Interestingly, there were two samples, MSK761 and MSK900b, that were assigned to ASCL1 or ASCL1/NEUROD1 groups by transcription factor expression, but which also clustered closer to POU2F3-driven PDXs by PCA and were notable for expression of *YAP1* and its downstream genes such as *LATS2* or *CAV1*. As mentioned earlier, the role of *YAP1* as a defining subtype marker in SCLC has recently been questioned, and a new subtype defined by an inflamed gene signature was proposed^12^. While MSK761 showed high expression of antigen-presenting genes, MSK900b did not. MSK761 was derived from a liver biopsy performed at the initial diagnosis. MSK761c, a CDX sample derived from the blood of the same patient after chemoimmunotherapy and combination immunotherapy, showed high expression of *ASCL1* by IHC which was further confirmed by RNA-seq. The high *YAP1* expression seen in the treatment-naïve MSK761 liver PDX was not found in the subsequent MSK761c CDX. Moreover, MSK761c did not cluster with MSK761 in the PCA, but with the other *ASCL1*-driven samples, further demonstrating the heterogeneity of tumor cell characteristics found in this patient. Whether these observed differences might be attributable to the site of origin of these samples is unclear. Setting this case aside, the transcriptional analysis did not reveal any major differences between treated and treatment-naïve samples. Bigger sample size and definition of gene expression signatures that correlate with disease progression and drug resistance are needed.

A primary strength of PDX models is the close linkage to the patient. PDX samples allow us to generate multiple models from patients at diagnosis and subsequently from different stages during treatment, establishing a powerful resource for the analysis of disease progression and shifting drug sensitivities. We anticipate our cohort of 46 PDX/CDX samples, derived from 33 donors, to be of substantial use to other research groups studying SCLC. This resource, and others like it, offers a powerful system to characterize SCLC biology and inform clinical research treatment strategies for patients with SCLC.

## Methods

### Patient samples

Patient samples for the generation of PDX models and subsequent analyses were collected with written informed consent from patients under protocols approved by the MSKCC Institutional Review Board/Privacy Board (IRB Protocols 12-245, 06-107, and 14-091). Metadata for each patient can be found in Supplementary Data 1. Tumor DNA extracted from formalin-fixed paraffin-embedded tissue or frozen and normal DNA was used for genomic sequencing with Memorial Sloan Kettering-Integrated Mutational Profile of Actionable Cancer Targets (MSK-IMPACT)^20^. After PicoGreen quantification and quality control by Agilent BioAnalyzer, 3–143 ng of DNA were used to prepare libraries using the KAPA Hyper Prep Kit (Kapa Biosystems KK8504) with eight cycles of PCR. About 50–500 ng of each barcoded library were captured by hybridization in equimolar pools of 7–26 samples using the IMPACT (Integrated Mutation Profiling of Actionable Cancer Targets) assay (Nimblegen SeqCap), designed to capture all protein-coding exons and select introns of 410 or 468 commonly implicated oncogenes, tumor suppressor genes, and members of pathways deemed actionable by targeted therapies. Captured pools were sequenced on a HiSeq 2000, HiSeq 2500, or HiSeq 4000 in a PE100 or PE125 run using the HiSeq SBS Kit v4, TruSeq Rapid SBS Kit – HS, HiSeq Rapid SBS Kit v2, or HiSeq 3000/4000 SBS Kit (Illumina) producing an average of 574X coverage per tumor and 418X per normal.

Three iterative versions of the MSK-IMPACT next-generation sequencing panel that screens for mutations, translocations, and copy number alterations were used across this study, consisting of 341, 410, or 468 cancer-associated genes^20^. For PDX/CDX samples, MuTect, Pindel, and VarDict were used to detect single nucleotide variants (SNVs) and insertions and deletions (Indels) (https://github.com/mskcc/roslin-variant/wiki/Roslin-Methods-v2.5). Copy number alterations (CAN) was called using the FACETS^50^ EM algorithm. For clinical samples, MuTect, Vardict, Pindel, and Somatic Indel Detector were used to detect single nucleotide variants (SNVs) and insertions and deletions (Indels)^51^, with variant allele frequency thresholds set to 5% for non-hotspot alterations and 2% for hotspot sites. Copy number alterations (CAN) were identified using a custom algorithm^20^. Structural variants were detected using Delly^52^. All identified variants were filtered against the patient-matched normal sample and manually reviewed to retain only true positive somatic calls.

### Patient-derived xenografts

All animal experiments were approved by the Memorial Sloan Kettering Cancer Center (MSKCC) Animal Care and Use Committee (IACUC Protocol 04-03-009) and mice were housed in accredited facilities under pathogen-free conditions. Female NOD.Cg-Prkdc<scid> Il2rg<tm1Wjl>/SzJ (Stock #: 005557) (6–8 weeks old) were obtained from Jackson Laboratory. PDX and CDX models were generated from primary tumors and whole blood samples as described previously^13^. Briefly, cells were injected s.c. in the flank of one mouse per model and subsequently passaged multiple times. Tumor size was measured using a caliper once to twice weekly and the following equation was used to calculate volume: V = π/6 × L × W^2^ (L length; W width). Tumors were harvested when the tumor volume reached 1500 mm^3^. Maximal tumor size was not exceeded.

### RNA-sequencing

Sample preparation for RNA-sequencing and subsequent analysis was performed as in ref. ^26^. Briefly, Illumina HiSeq instrument (4000 or equivalent; a 2x150 bp paired-end (PE)) according to manufacturer’s instructions was used for RNA-sequencing in collaboration with Genewiz.

### RNA-seq analysis

The output data (FASTQ files) are mapped to the target genome using the rnaStar aligner^53^ that maps reads genomically and resolves reads across splice junctions. We use the two-pass mapping method outlined in ref. ^54^ in which the reads are mapped twice. The first mapping pass uses a list of known annotated junctions from Ensemble. Novel junctions found in the first pass are then added to the known junctions and a second mapping pass is done (on the second pass the RemoveNoncanoncial flag is used). After mapping, we post-process the output SAM files using the PICARD tools to add read groups, AddOrReplaceReadGroups which in addition sort the file and coverts it to the compressed BAM format.

We then compute the expression count matrix from the mapped reads using HTSeq (www-huber.embl.de/users/anders/HTSeq) and one of several possible gene model databases. The raw count matrix generated by HTSeq are then be processed using the R/Bioconductor package DESeq (www-huber.embl.de/users/anders/DESeq) which is used to both normalize the full dataset and analyze differential expression between sample groups.

A PCA analysis was done using the top 500 highest variance genes and the first two PCA components were plotted. Heatmaps were generated using both the log-transformed absolute normalized intensity of counts and also of the *Z*-score of log normalized intensity over each gene. The *Z*-score heatmaps were plotted for specific genes sets and the samples were clustered using standard hierarchical clustering with the Manhattan distance metric and Ward.D linkage from the hclust function in R. The intensity heatmaps were generated using both the top differentially expressed genes or the top variable expression genes as measured by the gene standard deviation. A sample/sample correlation plot was done by computing the Spearman correlation of the top 100 most variable genes. The correlation matrix was plotted using the corrplot function from the R corrplot package using the hclust function with complete linkage to order the samples. See Supplementary Table 1 for a list of program versions used in RNA-seq analysis.

### Data files

- Human:

- GENOME: UCSC HG19
- GTF: gencode.v18.annotation

### Tissue microarray construction

For the immunohistochemical evaluation of PDX samples, tissue microarray blocks were constructed containing 3 × 1 mm cores per case. Slides from these blocks were subsequently stained with H&E and reviewed by a pathologist for quality assessment.

### Immunohistochemistry

Formalin-fixed paraffin-embedded tissues (standard paraffin blocks for donor samples and TMA blocks for PDX samples) were used to obtain 4 µm sections for immunohistochemistry (IHC). IHC was performed using standard immuno-peroxidase methods and manufacturer instructions (See Table). All slides were scanned in Aperio AT2 digital whole slide scanner and reviewed with Aperio ImageScope v12.4.3.7005 and/or under a light microscope by an experienced pathologist. Nuclear staining was considered positive for ASCL1, MYC, NEUROD1, and POU2F3; whereas cytoplasmic for synaptophysin, cytoplasmic and membranous for CD56, and nuclear ± cytoplasmic for YAP1. Percentage of positive cells and staining intensity (0/1 + /2 + /3+) were recorded. H scores were calculated by multiplying the percentage of positive cells by the staining intensity. For PDX samples, three independent cores per sample were stained for the indicated protein, and the *H*-score was derived from the average of the three cores. For clinical samples, one core per sample was stained for the indicated protein and *H*-score derived from this single core. See Supplementary Table 2 for a list of antibodies used for immunohistochemistry.

### Statistics and reproducibility

RNA-seq was performed once for 43 PDX samples and IHC was performed once for 37 PDX samples and 19 clinical samples. MSK-IMPACT was performed once for 42 PDX samples and 26 clinical samples. No statistical method to predetermine sample size was used. SCLC patient samples that were successfully engrafted were used without further selection criteria. The experiments were not randomized. The Investigators were not blinded to allocation during experiments and outcome assessment.

### Reporting summary

Further information on research design is available in the Nature Research Reporting Summary linked to this article.

## Supplementary information

Supplementary Information

Description of Additional Supplementary Information

Supplementary Data 1

Reporting Summary

## Data Availability

The Gene expression data generated in this study has been deposited in the Arrayexpress database under accession code E-MTAB-11230. The genomic sequencing data generated in this study underlying Figs. 2 and 5g are publicly available via the cBioPortal for cancer genomics https://www.cbioportal.org/study/summary?id=lung_pdx_msk_2021. The raw data were available under restricted access, access can be obtained by contacting the corresponding author Charles Rudin at rudinc@mskcc.org. The remaining data were available within the Article, Supplementary Information, or Source Data File. Source data are provided with this paper.

## Source data

Source data File
