# Supplementary material for: Genomic and transcriptomic analysis of a library of small cell lung cancer patient-derived xenografts
Source: Nat Commun. 2022 Apr 19;13:2144. doi: 10.1038/s41467-022-29794-4 (PMC9018685; doi:10.1038/s41467-022-29794-4)
Supplement: Supplementary file 1 — Supplementary Information [file 41467_2022_29794_MOESM1_ESM.pdf]

Genomic and transcriptomic analysis of a library of small cell lung cancer patient-derived xenografts

Caeser et al.

Supplementary Table 1 Program versions used in RNA-seq analysis

Program	Version
**HTSEQ**	htseq/HTSeq-0.5.3
**PICARD**	picard/picard-tools-1.124
**R**	R/R-3.2.0
**STAR**	star/STAR-STAR_2.5.0a
**SAMTOOLS**	samtools/samtools-0.1.19

Supplementary Table 2 Antibodies used for the immunohistochemical evaluation of PDX and clinical samples

Antibody	Clone	Species	Dilution	Manufacturer	Platform
**ASCL1**	24B72D11.11	Mouse (m)	1:100	BD	Leica Bond-RX
**CD56**	MRQ42	Rabbit (m)	1:100	Cell Marque	Leica Bond-RX
**MYC**	Y69	Rabbit (m)	1:250	Abcam	Leica Bond-RX
**NEUROD1**	EPR 17084	Rabbit (m)	1:50	Abcam	Leica Bond-RX
**POU2F3**	6D1	Mouse (m)	1:600	Santa Cruz	Leica Bond-RX
**Synaptophysin**	SNP88	Mouse (m)	1:1000	BioGenex	Ventana Discovery Ultra
**YAP1**	63.7	Mouse (m)	1:2000	Santa Cruz	Leica Bond-RX

(m): monoclonal

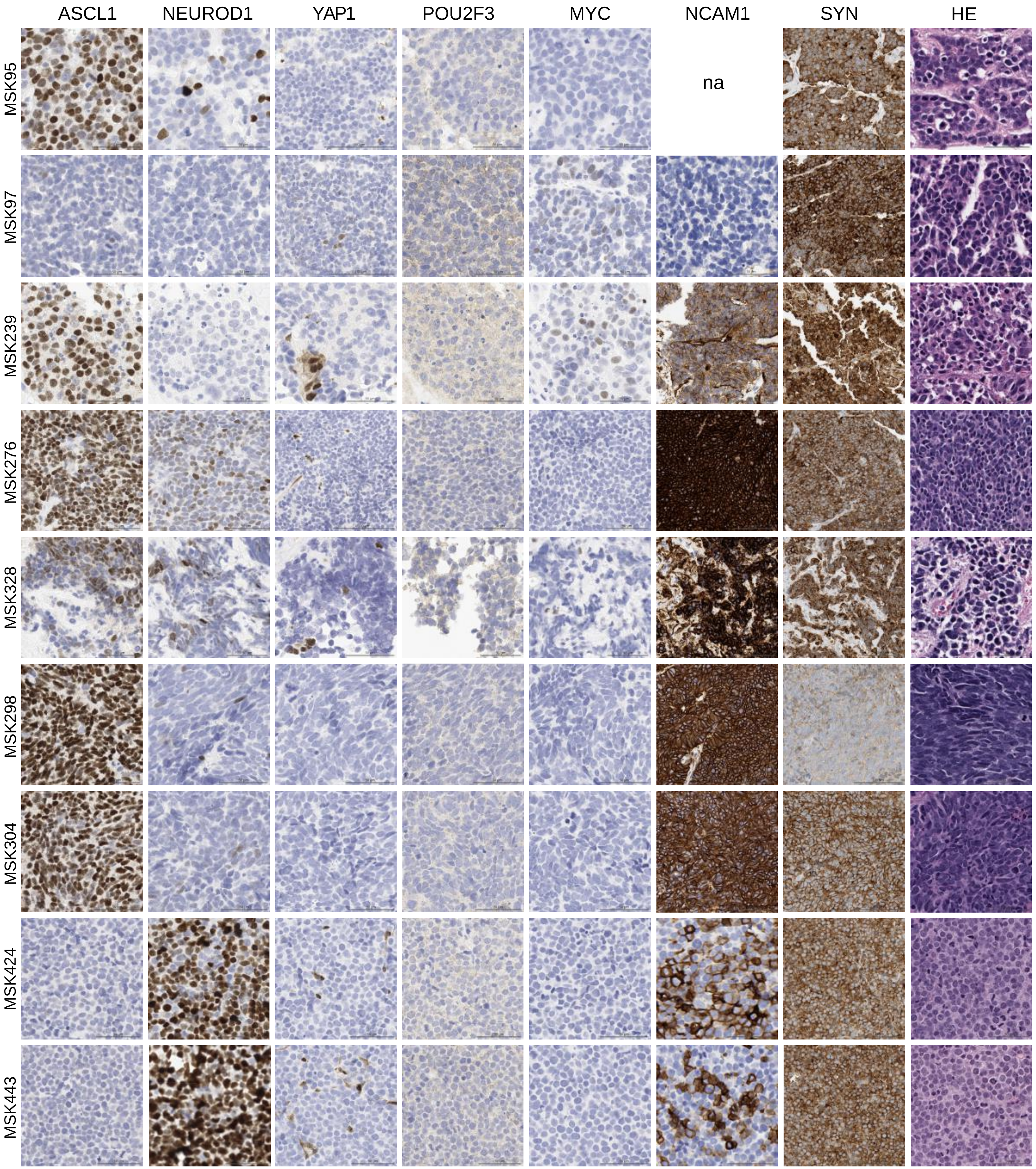

Supplementary Figure 1 Histologic analysis of PDX/CDX samples

H&E and immunohistochemistry images for the indicated proteins are shown for the 37 patient-derived/circulating tumor cell-derived xenograft (PDX/CDX) samples (9 samples shown here). Three independent cores per sample were stained for H&E and the indicated protein with a representative example shown here. Scale bar as indicated, 50μM.

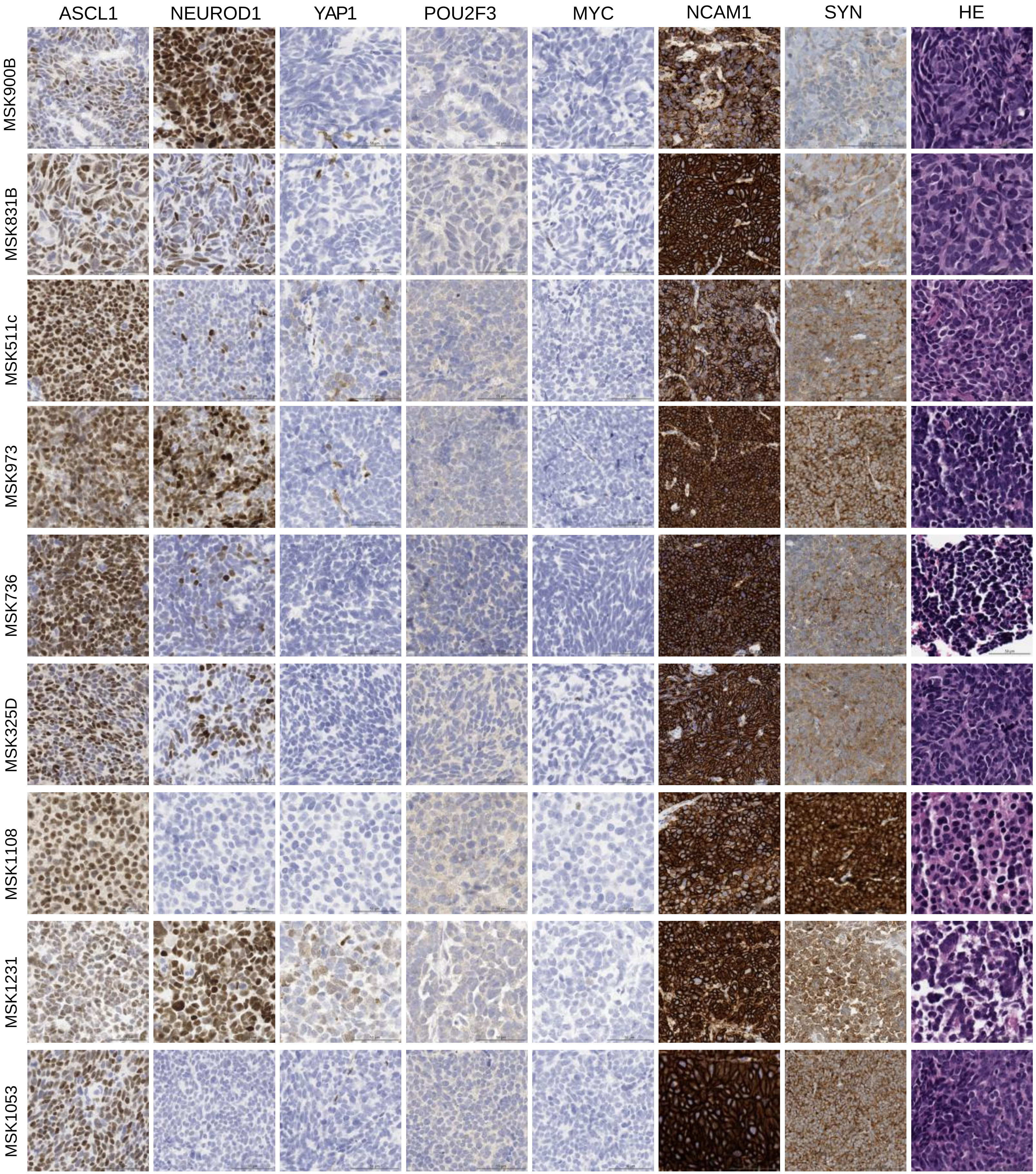

Supplementary Figure 2 Histologic analysis of PDX/CDX samples

H&E and immunohistochemistry images for the indicated proteins are shown for the 37 patient-derived/circulating tumor cell-derived xenograft (PDX/CDX) samples (9 samples shown here). Three independent cores per sample were stained for H&E and the indicated protein with a representative example shown here. Scale bar as indicated, 50μM.

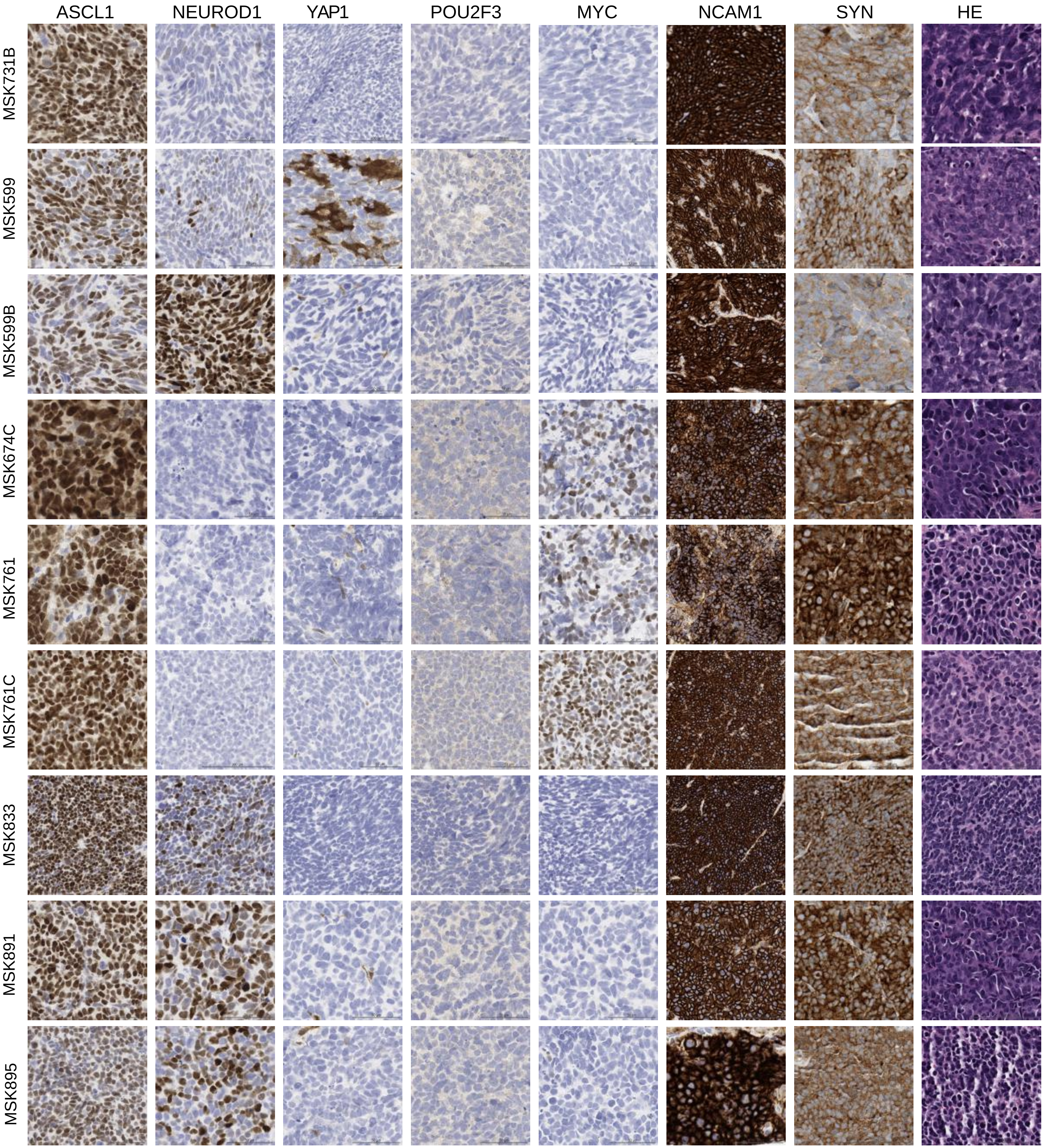

Supplementary Figure 3 Histologic analysis of PDX/CDX samples

H&E and immunohistochemistry images for the indicated proteins are shown for the 37 patient-derived/circulating tumor cell-derived xenograft (PDX/CDX) samples (9 samples shown here). Three independent cores per sample were stained for H&E and the indicated protein with a representative example shown here. Scale bar as indicated, 50μM.

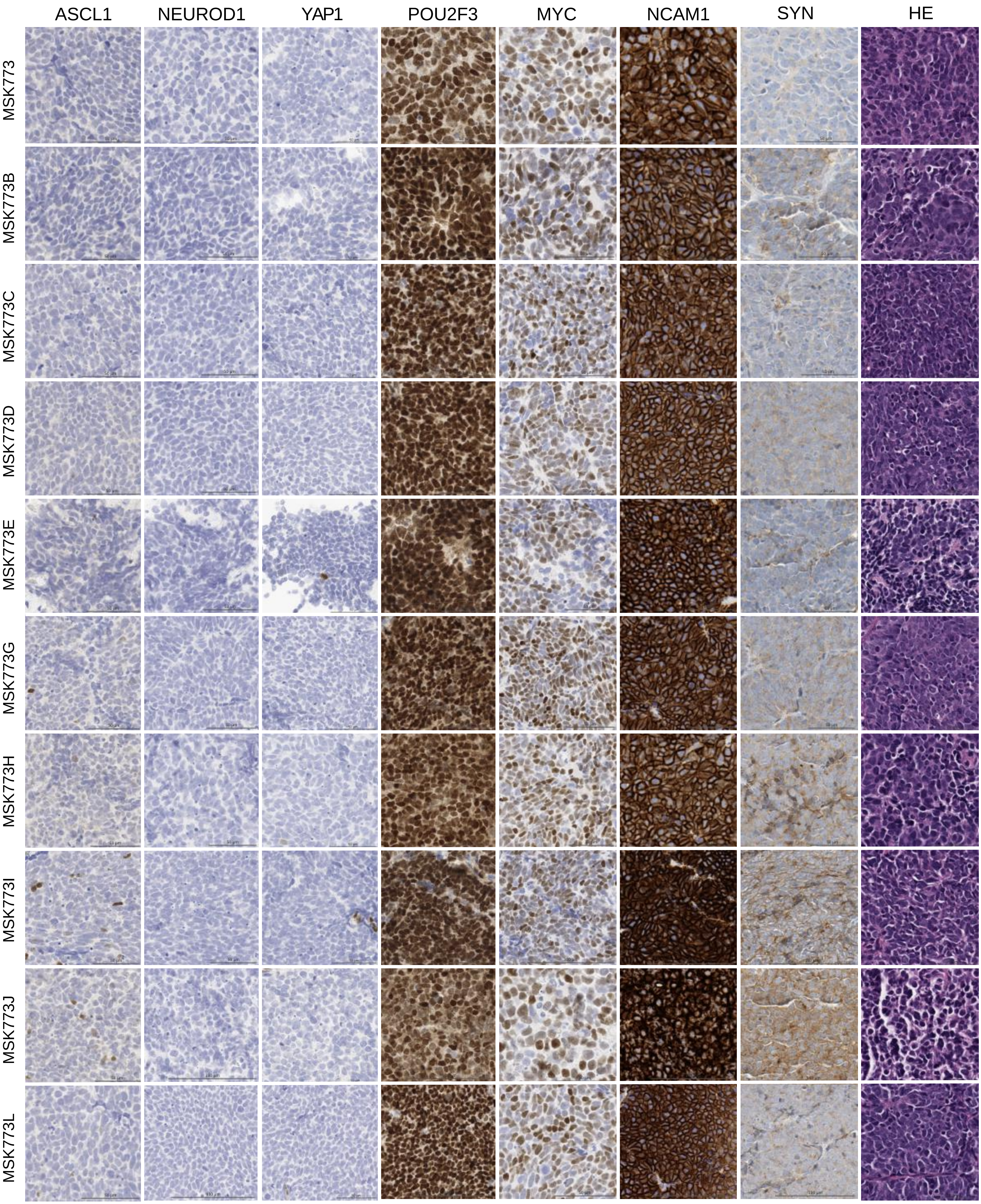

Supplementary Figure 4 Histologic analysis of PDX/CDX samples

H&E and immunohistochemistry images for the indicated proteins are shown for the 37 patient-derived/circulating tumor cell-derived xenograft (PDX/CDX) samples (10 samples shown here). Three independent cores per sample were stained for H&E and the indicated protein with a representative example shown here. Scale bar as indicated, 50μM.

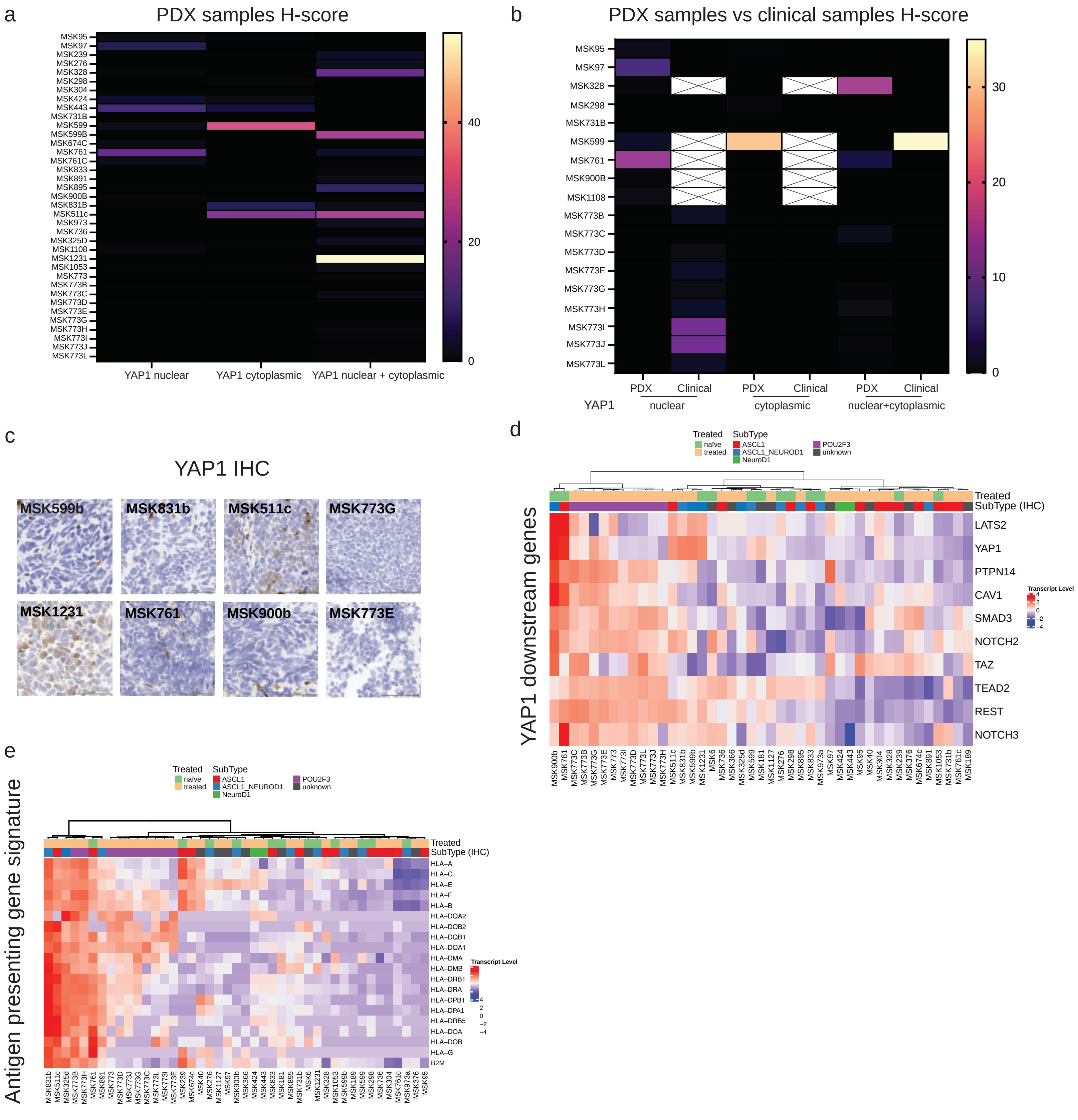

Supplementary Figure 5 YAP1 expression in PDX/CDX and clinical samples

a Heatmap depicts H-scores derived from 37 scored patient-derived xenograft (PDX) tumors for the fraction of positive cells and staining intensity, for YAP1 nuclear, cytoplasmic, and combined. Source data are provided as a Source Data file. **b** Heatmap depicts H-scores derived from 18 paired PDX tumors and clinical samples for the fraction of positive cells and staining intensity, for YAP1 nuclear, cytoplasmic and combined. White boxes with cross indicate scoring was not performed based on nuclear or cytoplasmic, only combined. Source data are provided as a Source Data file. **c** Immunohistochemistry images for YAP1 are shown for six selected PDX samples as indicated. Three independent cores per sample were stained for YAP1 with a representative example shown here. Scale bar as indicated, 50μM. **d** Heatmap of gene expression of 43 PDX samples for YAP1 downstream genes[30]. Color-coded panel on top depicts treatment status (naïve or treated) and subtype annotation based on IHC. Source data are provided as a Source Data file. **e** Heatmap of gene expression of 43 PDX samples for antigen presenting genes[12]. Color-coded panel on top depicts treatment status (naïve or treated) and subtype annotation based on IHC. Source data are provided as a Source Data file..

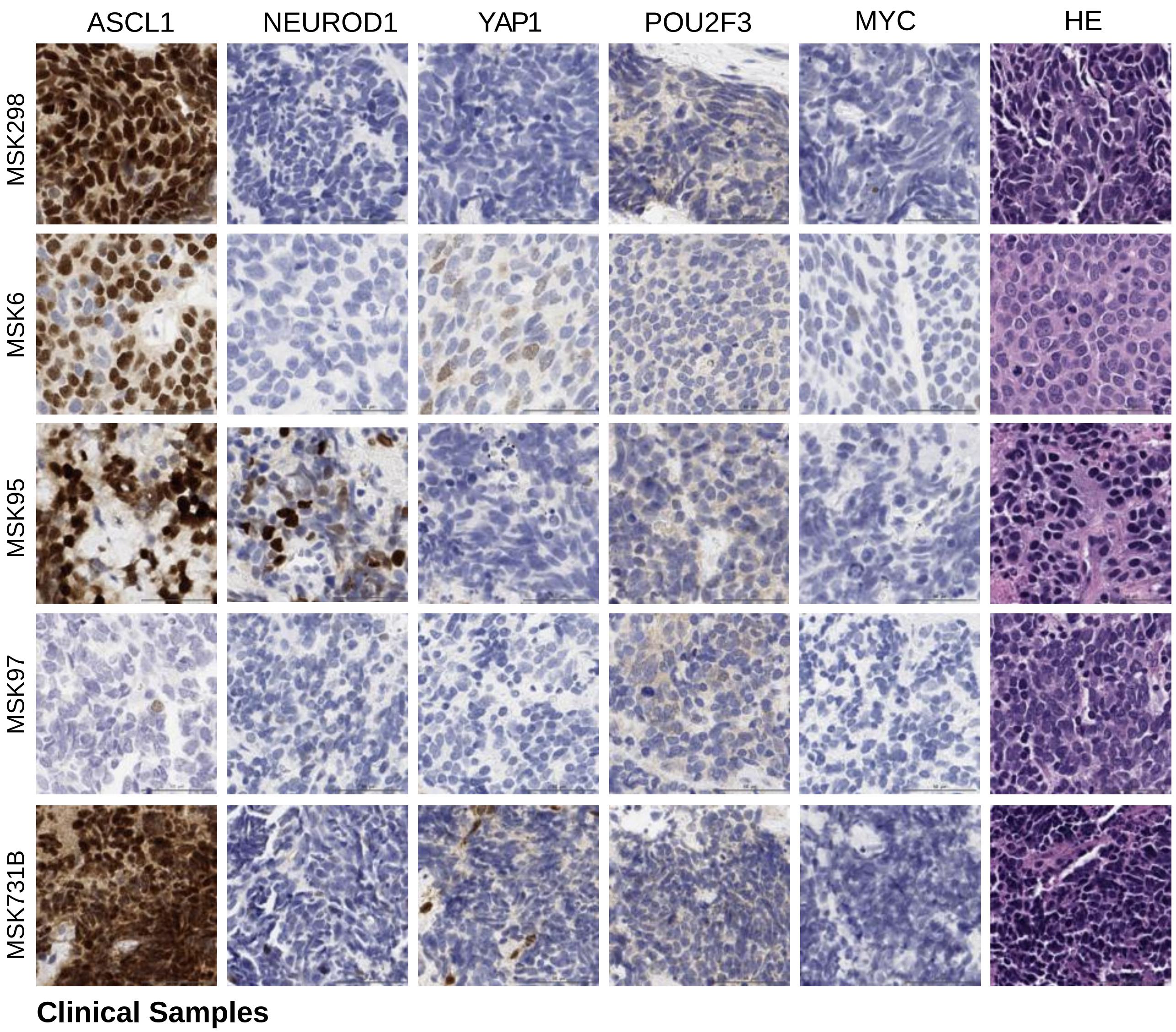

Supplementary Figure 6 Histologic analysis of clinical samples
H&E and immunohistochemistry images for the indicated proteins are shown for 14 clinical specimens (5 shown here). One core per sample was stained for H&E and the indicated protein with a representative example shown here. Scale bar as indicated, 50μM.

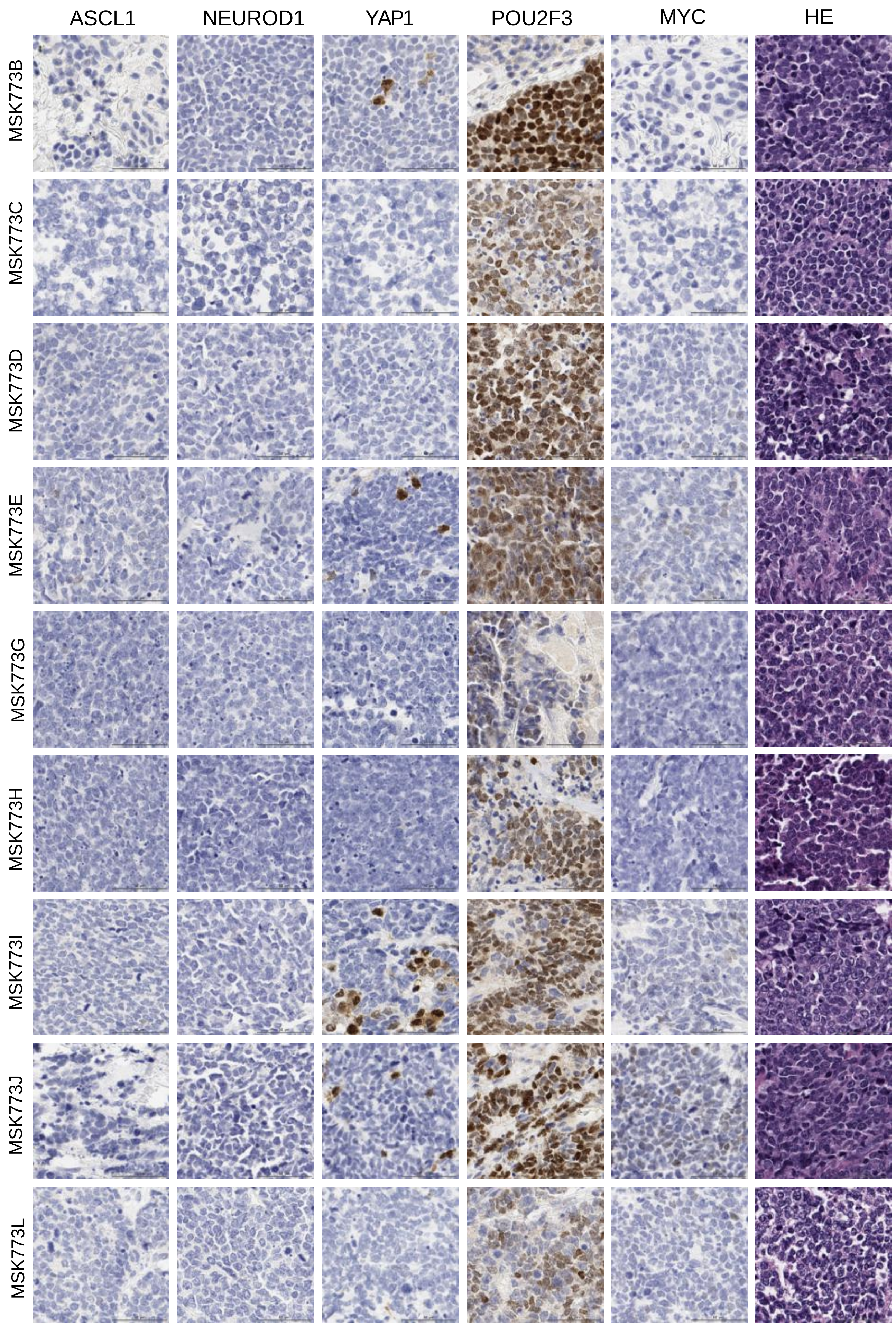

Supplementary Figure 7 Histologic analysis of clinical samples
H&E and immunohistochemistry images for the indicated proteins are shown for 14 clinical specimens (9 shown here). One core per sample was stained for H&E and the indicated protein with a representative example shown here. Scale bar as indicated, 50µM.

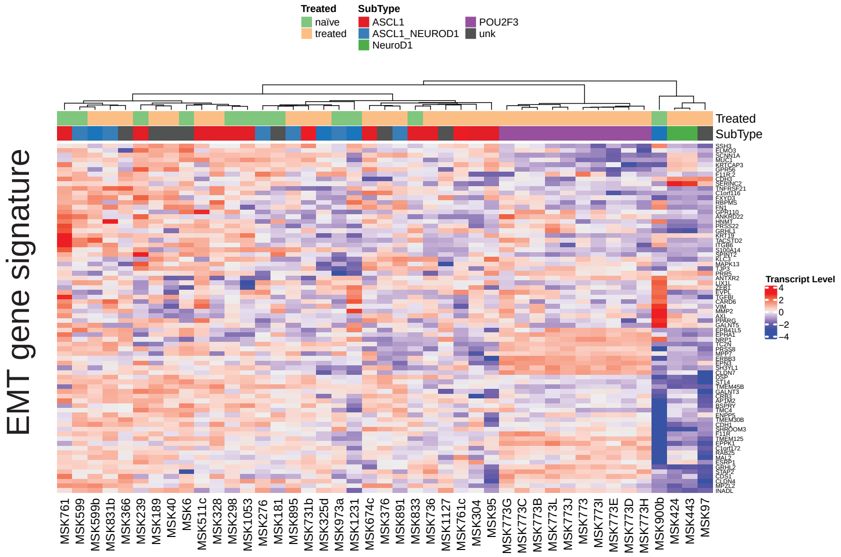

Supplementary Figure 8 EMT gene signature of PDX samples

Heatmap of gene expression of 43 patient-derived xenograft (PDX) samples for epithelial to mesenchymal transition (EMT) gene signature taken from34. Color-coded panel on top depicts treatment status (naïve or treated) and subtype annotation based on IHC. Source data are provided as a Source Data file.
