# Supplementary material for: Genomic and transcriptomic analysis of a library of small cell lung cancer patient-derived xenografts
Source: Nat Commun. 2022 Apr 19;13:2144. doi: 10.1038/s41467-022-29794-4 (PMC9018685; doi:10.1038/s41467-022-29794-4)
Supplement: Supplementary file 2 — Description of Additional Supplementary Information [file 41467_2022_29794_MOESM2_ESM.pdf]

Inventory of Supplementary Information

Supplementary Table 1 Program versions used in RNA-seq analysis

Supplementary Table 2 Antibodies used for the immunohistochemical evaluation of PDX and clinical samples

Supplementary Figure 1-4 Histologic analysis of PDX/CDX samples

Supplementary Figure 5 YAP1 expression in PDX/CDX and clinical samples

Supplementary Figures 6-7 Histologic analysis of clinical samples

Supplementary Figure 8 EMT gene signature of PDX samples
