# Supplementary material for: Genomic and transcriptomic analysis of a library of small cell lung cancer patient-derived xenografts
Source: Nat Commun. 2022 Apr 19;13:2144. doi: 10.1038/s41467-022-29794-4 (PMC9018685; doi:10.1038/s41467-022-29794-4)
Supplement: Supplementary file 4 — Reporting Summary [file 41467_2022_29794_MOESM4_ESM.pdf]

nature portfolio

Corresponding author(s): Charles M Rudin & Triparna Sen

Last updated by author(s): Feb 4, 2022

Reporting Summary

Nature Portfolio wishes to improve the reproducibility of the work that we publish. This form provides structure for consistency and transparency in reporting. For further information on Nature Portfolio policies, see our Editorial Policies and the Editorial Policy Checklist.

Statistics

For all statistical analyses, confirm that the following items are present in the figure legend, table legend, main text, or Methods section.

n/a	Confirmed	
☐	☒	The exact sample size (*n*) for each experimental group/condition, given as a discrete number and unit of measurement
☒	☐	A statement on whether measurements were taken from distinct samples or whether the same sample was measured repeatedly
☒	☐	The statistical test(s) used AND whether they are one- or two-sided<br>*Only common tests should be described solely by name; describe more complex techniques in the Methods section.*
☒	☐	A description of all covariates tested
☒	☐	A description of any assumptions or corrections, such as tests of normality and adjustment for multiple comparisons
☒	☐	A full description of the statistical parameters including central tendency (e.g. means) or other basic estimates (e.g. regression coefficient) AND variation (e.g. standard deviation) or associated estimates of uncertainty (e.g. confidence intervals)
☒	☐	For null hypothesis testing, the test statistic (e.g. *F*, *t*, *r*) with confidence intervals, effect sizes, degrees of freedom and *P* value noted<br>*Give P values as exact values whenever suitable.*
☒	☐	For Bayesian analysis, information on the choice of priors and Markov chain Monte Carlo settings
☒	☐	For hierarchical and complex designs, identification of the appropriate level for tests and full reporting of outcomes
☒	☐	Estimates of effect sizes (e.g. Cohen's *d*, Pearson's *r*), indicating how they were calculated

Our web collection on statistics for biologists contains articles on many of the points above.

Software and code

Policy information about availability of computer code

Data collection	Illumina HiSeq 4000
Data analysis	HTSEQ (Version htseq/HTSeq-0.5.3) , PICARD (Version picard/picard-tools-1.124), R (Version R/R-3.2.0), STAR (Version star/STAR-STAR_2.5.0a), SAMTOOLS (Version samtools/samtools-0.1.19)

For manuscripts utilizing custom algorithms or software that are central to the research but not yet described in published literature, software must be made available to editors and reviewers. We strongly encourage code deposition in a community repository (e.g. GitHub). See the Nature Portfolio guidelines for submitting code & software for further information.

Data

Policy information about availability of data

All manuscripts must include a data availability statement. This statement should provide the following information, where applicable:

- Accession codes, unique identifiers, or web links for publicly available datasets
- A description of any restrictions on data availability
- For clinical datasets or third party data, please ensure that the statement adheres to our policy

The Gene expression data generated in this study has been deposited in the Arrayexpress database under accession code E-MTAB-11230 [https://www.ebi.ac.uk/arrayexpress/experiments/E-MTAB-11230/]. Full MSK-IMPACT profiling of each model including mutational and copy number data is available at https://www.cbioportal.org/study/summary?id=lung_pdx_msk_2021.
All the other data supporting the findings of this study are available within the article and its supplementary information files. Source data are provided with this paper.

Field-specific reporting

Please select the one below that is the best fit for your research. If you are not sure, read the appropriate sections before making your selection.

☒ Life sciences ☐ Behavioural & social sciences ☐ Ecological, evolutionary & environmental sciences

For a reference copy of the document with all sections, see nature.com/documents/nr-reporting-summary-flat.pdf

Life sciences study design

All studies must disclose on these points even when the disclosure is negative.

Sample size	At the time of writing this manuscript, 46 samples from 33 patients were available for analysis. No statistical method to predetermine sample size was used. Sample size is based purely on what was available at the time. Complete overview of sample size can be found in Figure 1A in the Manuscript.
Data exclusions	SCLC patient samples that successfully engrafted were used without further selection criteria.
Replication	Cell number obtained from patients is limited so study was not replicated.
Randomization	The experiments were not randomized.
Blinding	The Investigators were not blinded to allocation during experiments and outcome assessment

Reporting for specific materials, systems and methods

We require information from authors about some types of materials, experimental systems and methods used in many studies. Here, indicate whether each material, system or method listed is relevant to your study. If you are not sure if a list item applies to your research, read the appropriate section before selecting a response.

Materials & experimental systems

n/a	Involved in the study	
☐	☒	Antibodies
☒	☐	Eukaryotic cell lines
☒	☐	Palaeontology and archaeology
☐	☒	Animals and other organisms
☐	☒	Human research participants
☒	☐	Clinical data
☒	☐	Dual use research of concern

Methods

n/a	Involved in the study	
☒	☐	ChIP-seq
☒	☐	Flow cytometry
☒	☐	MRI-based neuroimaging

Antibodies

Antibodies used

Antibody Clone Species Dilution Manufacturer Platform Cat No
ASCL1 24B72D11.11 Mouse (m) 1:100 BD Leica Bond-RX Cat No 556604
CD56 MRQ42 Rabbit (m) 1:100 Cell Marque Leica Bond-RX Cat No 156R-96
MYC Y69 Rabbit (m) 1:250 Abcam Leica Bond-RX Cat No 32072
NEUROD1 EPR 17084 Rabbit (m) 1:50 Abcam Leica Bond-RX Cat No 205300
POU2F3 6D1 Mouse (m) 1:600 Santa Cruz Leica Bond-RX Cat No sc293402
Synaptophysin SNP88 Mouse (m) 1:1000 BioGenex Ventana Discovery Ultra Cat No MU363-UC
YAP1 63.7 Mouse (m) 1:2000 Santa Cruz Leica Bond-RX Cat No sc101199

All antibodies used for IHC in this study are commercially available and have been validated by the manufacturer.

Validation

All antibodies used for IHC in this study are commercially available and have been validated by the manufacturer.

Animals and other organisms

Policy information about studies involving animals; ARRIVE guidelines recommended for reporting animal research

Laboratory animals	Female NOD.Cg-Prkdc<scid> Il2rg<tm1Wjl>/SzJ (Stock #: 005557) (6-8 weeks old) were obtained from Jackson Laboratory.
Wild animals	No wild animals were utilized during the course of these studies.

Field-collected samples	No field-collected samples were utilized during the course of these studies.
Ethics oversight	All animal experiments were approved by the Memorial Sloan Kettering Cancer Center (MSKCC) Animal Care and Use Committee and mice were housed in accredited facilities under pathogen-free conditions. PDX Protocol 14-091.

Note that full information on the approval of the study protocol must also be provided in the manuscript.

Human research participants

Policy information about studies involving human research participants

Population characteristics	Metadata for each patient can be found in Supplementary Table 1 in the Manuscript.
Recruitment	No self-selection bias present, patient samples that were included in this manuscript was based purely on SCLC diagnosis. Whenever material from SCLC patient is available, it gets engrafted into NSG mice to create PDX models.
Ethics oversight	46 samples from the 33 patients reported in this study were collected with signed informed consent from patients. All animal experiments were approved by the Memorial Sloan Kettering Cancer Center (MSKCC) Animal Care and Use Committee (MSK IMPACT Protocol 12-245; PDX Protocol 14-091; Biospecimen Protocol 06-107).

Note that full information on the approval of the study protocol must also be provided in the manuscript.
